# An exploration of needs and preferences for dietary support in colorectal cancer survivors: A mixed-methods study

**DOI:** 10.1371/journal.pone.0189178

**Published:** 2017-12-18

**Authors:** Meeke Hoedjes, Anja de Kruif, Floortje Mols, Martijn Bours, Sandra Beijer, Renate Winkels, Marjan J. Westerman, Jaap C. Seidell, Ellen Kampman

**Affiliations:** 1 Department of Health Sciences and the EMGO+ Institute for Health and Care Research, VU University Amsterdam, Amsterdam, the Netherlands; 2 Center of Research on Psychology in Somatic diseases, Department of Medical and Clinical Psychology, Tilburg University, Tilburg, the Netherlands; 3 Division of Human Nutrition, Wageningen University, Wageningen, the Netherlands; 4 Netherlands Comprehensive Cancer Organisation (IKNL), Utrecht, the Netherlands; 5 Department of Epidemiology, GROW-School for Oncology and Developmental Biology, Maastricht University, Maastricht, the Netherlands; McMaster University, CANADA

## Abstract

**Purpose:**

To describe the proportion of colorectal cancer (CRC) survivors who perceive a need for dietary support; to examine which socio-demographic, cancer-related, and health-related characteristics are associated with this need; to explore reasons for (not) needing support; and to explore CRC survivors’ specific needs and preferences with regard to lifestyle (i.e., dietary, exercise, and/or weight management) support.

**Methods:**

This mixed-methods study comprised a cross-sectional survey among 1774 Dutch CRC survivors and three focus groups (n = 16). To examine associations, logistic regression analyses were conducted. Focus groups were audio-taped, transcribed verbatim, and analyzed using a thematic approach.

**Results:**

Of 1458 respondents (82%), 1198 (67.5%) were included for analyses. 17.5% reported a need for dietary support. Characteristics associated with this need were: being younger, living without a partner, having a stoma, having diabetes, and being overweight or obese. The main reason for needing support was being unable to initiate and maintain lifestyle changes without support. CRC survivors preferred receiving information soon after diagnosis to make an autonomous, informed decision on improving their lifestyle. They preferred to receive individually-tailored lifestyle support in an autonomy-supportive environment, preferably with involvement of their family and fellow-sufferers.

**Conclusions:**

This study has provided knowledge on appropriate support for CRC survivors in need for dietary support to improve health outcomes by promoting adherence to lifestyle and body weight recommendations. Findings can be used to better identify CRC survivors in need for dietary support, and to tailor lifestyle support to their needs and preferences in order to promote uptake, adherence, and effectiveness.

## Introduction

Colorectal cancer (CRC) survivors are encouraged to meet lifestyle (i.e., dietary, physical activity[1]) and body weight recommendations[2–4] to increase their quality of life, and to decrease their risk for cardiovascular disease, diabetes mellitus type II, and mortality[5–8]. However, it has been shown that the majority do not meet these recommendations[9–14]. For example, a recent survey among CRC survivors showed that most did not meet recommendations on consumption of plant-based foods (91%) and red and processed meat (92%)[14]. In addition, about two-thirds did not meet the recommendation on body fatness[13, 14]. These findings suggest a need for improvement of lifestyle behaviors among CRC survivors[4, 15].

To promote adherence to lifestyle and body weight recommendations among CRC survivors, lifestyle support (i.e., dietary, exercise, and/ or weight management support) can be offered. Dietary support is particularly relevant for CRC survivors, because of the potential beneficial effects of dietary adaptations on frequently reported bowel complaints related to CRC and its treatment, such as diarrhoea and intolerance of certain foods[16]. However, such support is currently not routinely provided by oncology health care professionals. Although receiving a cancer diagnosis has been marked as a ‘teachable moment’ to promote adherence to lifestyle and body weight recommendations[17], research has shown that CRC survivors perceive a lack of information on lifestyle and body weight recommendations after diagnosis[18–21]. Since receiving information or advice is commonly not sufficient to be able to improve lifestyle for those in need of support, additional behavioural support is needed (e.g., behavioural counselling, including the use of behaviour change techniques such as goal setting, action planning, and review of behaviour goals) [22]. A first step towards offering appropriate support, is to conduct a needs assessment to gain more insight into the target population[23]. To our knowledge, the proportion of CRC survivors who perceive a need for dietary support has not previously been evaluated and their characteristics have not been assessed. Hence, it is currently unknown which CRC survivors particularly perceive a need for dietary support. Knowledge on reasons for (not) needing support would provide additional insight into factors influencing uptake of such support, and may be used to promote uptake. To promote uptake, adherence to, and effectiveness of support, it is important that the support that is offered to CRC survivors fits their needs and preferences. To date, there is limited knowledge on CRC survivors’ needs and preferences with regard to such support[19, 24, 25].

Therefore, the aims of this study were: 1) to describe the proportion of CRC survivors who perceive a need for dietary support; 2) to examine which socio-demographic, cancer-related, and health-related characteristics are associated with this need; 3) to explore reasons for (not) needing support; and 4) to explore specific needs and preferences with regard to lifestyle (i.e., dietary, exercise, and/or weight management) support.

## Methods

A mixed-methods design was used to explore needs and preferences for dietary support among CRC survivors. A cross-sectional survey was conducted to describe what proportion of CRC survivors perceived a need for dietary support and to examine which characteristics were associated with this need. In addition, focus groups were conducted to explore CRC survivors’ reasons for (not) needing lifestyle support and their specific needs and preferences with regard to lifestyle support.

### Survey

#### Setting and study population

The cross-sectional survey was part of a larger, population-based prospective observational survey among CRC survivors. Details on this longitudinal study can be found elsewhere[20]. Briefly, all CRC patients diagnosed between January 2000 and June 2009 were sampled from the southern area of the Netherlands Cancer Registry (NCR). The NCR contains clinical data on all newly diagnosed cancer patients in the Netherlands. After the initial patient selection, the Patient Reported Outcomes Following Initial Treatment and Long term Evaluation of Survivorship (PROFILES) registry was used for data collection (http://www.profilesregistry.nl)[26]. See van de Poll-Franse et al (2011) for more information on the NCR, the PROFILES registry, and their interrelation [26]. Patients with cognitive impairments, unverifiable addresses and patients who died prior to the study start were excluded. From 2010 onwards, yearly surveys were performed by means of self-administered questionnaires. In the present study, cross-sectional data from the third survey in December 2012 are presented. An item on the need for dietary support was added to this third survey. The study was approved by the Medical Ethical Research Committee of the Máxima Medical Center (ethics approval number 0822).

#### Data collection

A total of 1774 eligible CRC patients were invited for participation via a letter from their (ex-) attending specialist. Participants were asked to complete an online or a paper version of the questionnaire. After two months, a reminder with a paper questionnaire was sent. Patients were reassured that nonparticipation had no consequences for their follow-up care or treatment. Written informed consent was obtained from all participants.

#### Outcome measures

The need for dietary support was assessed in the survey by the statement “I feel the need for support to be able to eat healthier” (disagree/ agree). The need for dietary support to be able to eat healthier implies a need for behavioural counselling aimed at self-regulation of dietary behaviour to be able to adhere to dietary advice, rather than provision of dietary advice or information alone.

Age (at the time of recruitment), gender and clinical information including cancer diagnosis date, tumor site, primary cancer treatment and cancer stage were derived from the NCR. Socio-economic status (SES) was based on fiscal data on the national economic value of residences and household income aggregated per postal code[27]. Data on marital status and received follow-up care after cancer treatment at the time of the survey were self-reported. Having a stoma was assessed by an item from the CRC-specific module of the European Organization for Research and Treatment of Cancer Quality of Life Questionnaire (EORTC QLQ-CR38)[28]. ‘Having diabetes at the moment of the survey or during the 12 months before’ (yes/ no) was measured with the Self-Administered Comorbidity Questionnaire (SCQ)[29]. Self-reported body height (cm) and weight (kg) were used to calculate Body Mass Index (BMI). BMI was categorised as underweight (BMI < 18.5 kg/m2), normal weight (18.5 ≤ BMI < 25 kg/m2), overweight (25 ≤ BMI < 30 kg/m2), or obesity (BMI ≥ 30 kg/m2).

#### Statistical analyses

Statistical analyses were performed using IBM SPSS Statistics for Windows, Version 20. The population for analysis (n = 1198) consisted of participants with complete data on the variable ‘need for dietary support’ (yes/no). Differences between the population for analysis and those excluded from the population for analysis were tested using Independent Student’s t-tests for continuous variables and Chi-square tests for categorical variables. Mean and frequency tables were used to describe socio-demographic, cancer-related, and health-related characteristics.

Logistic regression analyses were used to examine which characteristics were associated with the need for dietary support. Univariate logistic regression analyses were conducted, with the need for dietary support (yes/ no) as dependent variable and one socio-demographic, cancer-related, or health-related characteristic as independent variable. The following socio-demographic characteristics were included in these analyses: mean age (at the time of recruitment in years), sex (male/female), marital status (with partner/ without partner), and SES (low/ medium/ high). Cancer-related characteristics included: tumour site (colon/ rectum), tumour stage (I-IV), mean time after diagnosis (in years), receiving follow-up care (yes/ no), radiotherapy (yes/ no), chemotherapy (yes/ no), and having a stoma (yes/ no). Health-related characteristics included: having diabetes (yes/no) and BMI. The BMI categories underweight and normal weight were combined for the statistical analyses, because only four CRC survivors were underweight.

Zero-order correlations were calculated to explore associations between the need for dietary support and each socio-demographic, cancer-related, or health-related characteristic, and to assess for multicollinearity between characteristics. These correlations showed that multicollinearity was an issue for the characteristics ‘radiotherapy’ and ‘tumour site’, since these characteristics were interrelated with correlations of >0.70[30]. A multiple logistic regression analysis was conducted, with the ‘need for dietary support’ as dependent variable, and all socio-demographic, cancer-related, or health-related characteristics, except for ‘radiotherapy’, as independent variables.

### Focus groups

#### Participants and procedure

Focus group participants were recruited from the COLON-study, an ongoing, multi-centre prospective cohort study among CRC survivors[31]. Overweight and obese CRC survivors were invited to participate in the focus groups, since the results of the logistic regression analyses showed that overweight and obese CRC survivors particularly perceived a need for dietary support. To ensure that participants were still overweight at the time of recruitment, only CRC survivors with a BMI ≥ 27 kg/m2 at 6 months after diagnosis were invited for participation in the focus groups. Because it was expected that CRC survivors were more willing and able to make sustainable lifestyle changes after recovery from treatment, only those diagnosed at least one year ago were invited to participate.

In total, 59 COLON-study participants were eligible and invited to participate in one of the three focus groups in July and December 2014. Since additional analyses showed that socio-demographic, cancer-related, and health-related characteristics differed between colon and rectal cancer survivors (data not shown), participants were purposively sampled based on tumour site.

#### Data collection

The focus groups were guided by a topic list based on sensitizing concepts, and included topics on: the perception of a healthy lifestyle; determinants of a healthy lifestyle and lifestyle change; readiness for lifestyle change, the need for lifestyle support; reasons for needing or not needing lifestyle support; and preferences for lifestyle support. The focus groups were audio-taped and lasted about two and a half hours each. Focus groups were moderated by a qualitative researcher (AdK), and observed by an accompanying researcher (MH).

#### Data analysis

Focus groups were transcribed verbatim, and transcripts were supplemented with field notes taken by the observer. Each focus group was discussed between the moderator and the observer. A thematic analysis was carried out to analyse the data by performing several phases of coding[32, 33]. During the first phase of open coding, the data was fragmented and coded. The second phase of axial coding focused on describing and ordering the codes. Finally, during the third phase of selective coding, the main themes were determined and categorized. Trends and patterns, and similarities and differences across the focus groups were identified. Furthermore, emergent themes were identified and overlapping clusters of information were combined, such that the themes were further refined and linked to the research question. To perform the coding, the paper-pencil method was applied. To increase validity of the study, the transcripts were coded by one researcher (MH) and the codes were checked and adjusted by another researcher (AdK) until consensus was reached. Analyses were partly performed concurrently with data collection so that results from the first focus group could be taken into account in the second and third focus group.

## Results

### Survey

**Fig 1:**
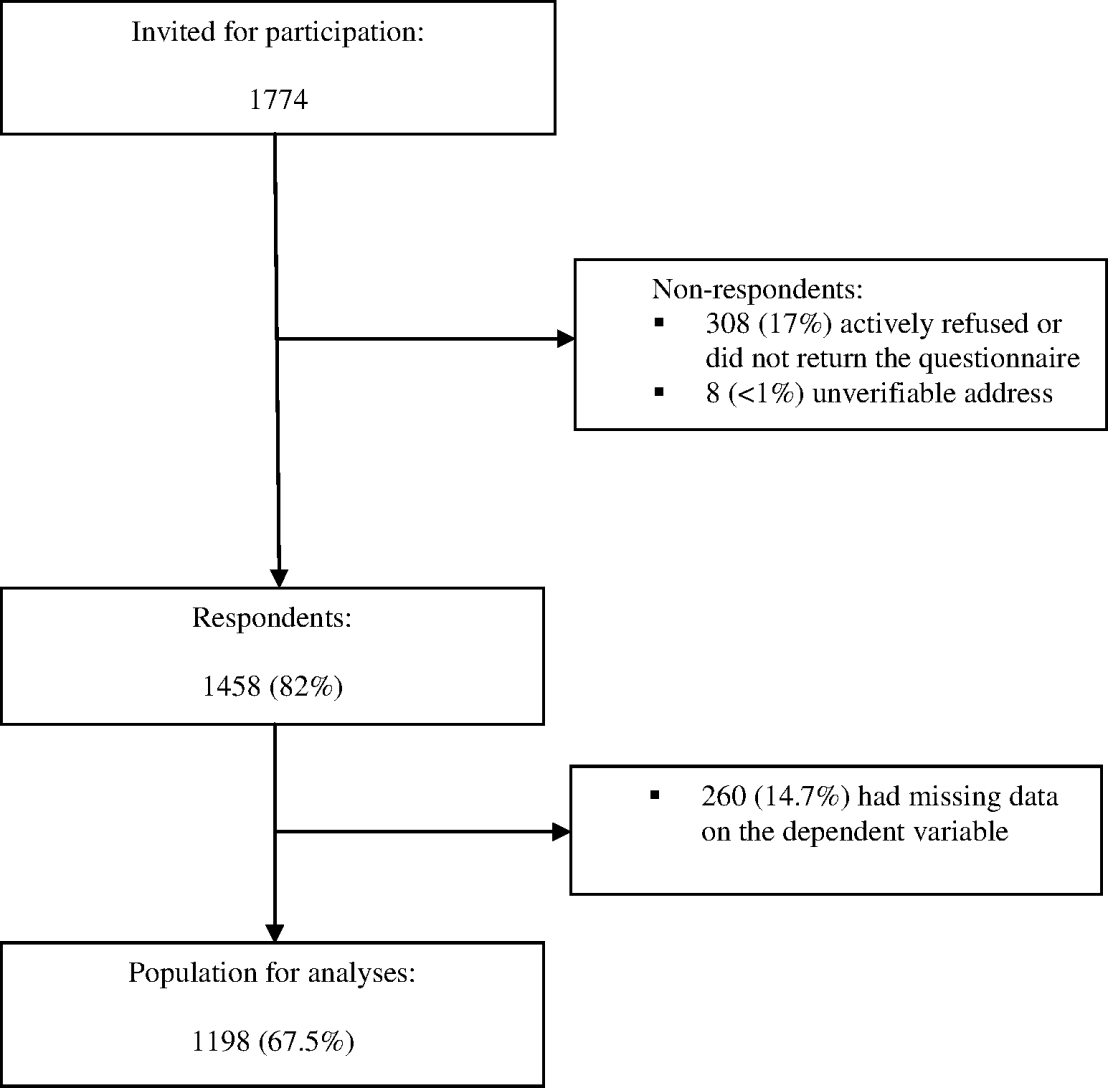
Flow-chart of study participants. For the present study, cross-sectional data from a larger longitudinal study among colorectal cancer survivors are presented. A flow diagram of participants in this longitudinal study has been published elsewhere[20]. The present study involves data obtained from survey 3 in December 2012.

Of the 1774 CRC survivors who were invited to participate in the survey, 1458 returned the questionnaire (response rate 82%). 1198 (67.5%) CRC survivors were included in the population for analysis. Non-respondents (n = 316) and respondents with missing data on the dependent variable (n = 260) were excluded (Fig 1). Compared with those who were excluded, those who were included were younger, more often male, had a higher SES, and were more often diagnosed with rectal cancer (all *p*<0.05; Table 1).

**Table 1:** Socio-demographic and cancer-related characteristics of colorectal cancer survivors who were invited to participate in the current study (n = 1774), and of those who were included (n = 1198) vs. excluded (n = 576) from population for analyses^1^.

	Total	Included	Excluded	p-value
	N = 1774	N = 1198 (67.5%)	N = 576 (32.5%)	
*Socio-demographic characteristics*				
Age at recruitment in years				
Mean (SD)	70.0(9.5)	69.1(9.5)	72.0(9.2)	**0.000**
Female [n(%)]	764(43.1)	479(40.0)	285(49.5)	**0.000**
SES [n(%)]				**0.001**
Low	333(19.6)	196(17.2)	137 (24.6)	
Medium	691(40.7)	468(41.0)	223(40.0)	
High	674(39.7)	477(41.8)	197(35.4)	
*Cancer-related characteristics*				
Tumour site [n(%)]				**0.046**
Colon	1068(60.2)	702(58.6)	366(63.5)	
Rectum	702(39.8)	496(41.4)	210(36.5)	
Tumour stage at diagnosis [n(%)]				0.503
Stage I	531(30.7)	364(31.4)	167(29.5)	
Stage II	632(36.6)	412(35.5)	220(38.8)	
Stage III	512(29.6)	351(30.3)	161(28.4)	
Stage IV	52(3.0)	33(2.8)	19(3.4)	
Time since diagnosis in years				
Mean(SD)	6.87(2.78)	6.88(2.8)	6.86(2.8)	0.919
Median(IQR)	6.07(5)			
Treatment [n(%)]				*
Surgery only	820(46.3)	549(45.9)	271(47.0)	
Surgery + radiotherapy	419(23.6)	294(24.6)	125(21.7)	
Surgery + chemotherapy	379(21.4)	249(20.8)	130(22.6)	
Surgery + radiotherapy + chemotherapy	147(8.3)	102(8.5)	45(7.8)	
Chemotherapy only	4(0.2)	2(0.2)	2(0.3)	
Chemotherapy + radiotherapy	3(0.2)	0	3(0.5)	

^1^ The population for analyses consists of participants with complete data on the dependent variable ‘need for dietary support’ (yes = 1; no = 0). Non-respondents (n = 316) as well as respondents with missing data on the dependent variable (n = 260) were excluded from the population for analyses.

Abbreviations: SD = Standard Deviation; IQR = InterQuartile Range; SES = Socio-Economic Status

*Chi-square cannot be calculated since 4 cells (33.3%) have an expected count of less than 5. However, separate chi-square tests for surgery, chemotherapy, and radiotherapy revealed that those included did not differ from those excluded from the population for analyses with regard to treatment. (Data not shown)

A p-value of <0.05 was considered to be statistically significant. Statistically significant p-values are printed bold.

#### Need for dietary support and associated characteristics

Of 1198 CRC survivors, 17.5% reported a need for dietary support to be able to eat healthier. The univariate logistic regression analyses showed that CRC survivors with a need for dietary support were younger (OR 0.98; 95% CI: 0.97–0.99) and more often living without a partner (OR 1.54; 95% CI: 1.09–2.17), and more often had a stoma (OR 1.46; 95% CI: 1.03–2.09), diabetes (OR 2.33; 95% CI: 1.57–3.47), and a higher BMI (OR 1.11; 95% CI: 1.07–1.14) compared with CRC survivors without a need for dietary support. The multivariable logistic regression analysis showed comparable results, except for the variable ‘having a stoma’, which was no longer statistically significantly associated with the need for dietary support (Table 2).

**Table 2:** Socio-demographic, cancer-related, and health-related characteristics of colorectal cancer survivors (n = 1198) and associations with the need for dietary support.

	Total N = 1198	Need for support N = 210	No need for support N = 988	Univariate^1^	Multivariable^2^
	N(%) Unless otherwise specified	N(%) Unless otherwise specified	N(%) Unless otherwise specified	OR(95%CI)	OR(95%CI)
*Socio-demographic characteristics*					
Age [mean(SD)]	69.1(9.5)	67.7(10.7)	69.3(9.2)	**0.98(0.97–0.99)**	**0.97(0.95–0.99)**
Sex					
Male	719(60.0)	117(55.7)	602(60.9)	0.81(0.60–1.10)	0.81(0.54–1.21)
Female	479(40.0)	93(44.3)	386(39.1)	1	1
Marital status					
Living with a partner	261(21.9)	148(71.5)	782(79.5)	1	1
Living without a partner	930(78.1)	59(28.5)	202(20.5)	**1.54(1.10–2.17)**	**1.84(1.16–2.92)**
SES					
Low	196(17.2)	42(20.7)	154(16.4)	1.30(0.86–1.96)	0.85(0.49–1.45)
Medium	468(41.0)	78(38.4)	390(41.6)	0.95(0.68–1.33)	0.72(0.48–1.10)
High	477(41.8)	83(40.9)	394(42.0)	1	1
*Cancer-related characteristics*					
Tumour site					
Colon	702(58.6)	115(54.8)	587(59.4)	0.83(0.61–1.12)	0.90(0.58–1.40)
Rectum	496(41.4)	95(45.2)	401(40.6)	1	1
Tumour stage at diagnosis					
Stage I	364(31.4)	67(33.2)	297(31.0)	1	1
Stage II	412(35.5)	70(34.7)	342(35.7)	0.91(0.63–1.31)	1.00(0.62–1.62)
Stage III	351(30.3)	61(30.2)	290(30.3)	0.93(0.64–1.37)	0.97(0.54–1.74)
Stage IV	33(2.8)	4(2.0)	29(3.0)	0.61(0.21–1.80)	0.75(0.22–2.55)
Time since diagnosis in years [Mean(SD)]	6.9(2.8)	6.9(2.6)	6.9(2.8)	1.00(0.95–1.06)	1.02(0.95–1.10)
Receiving follow-up care					
No	305(25.8)	54(26.2)	251(25.7)	1	1
Yes	877(74.2)	152(73.8)	725(74.3)	0.98(0.69–1.37)	0.90(0.57–1.43)
Radiotherapy					
No	802(66.9)	140(66.7)	662(67.0)	1	^3^
Yes	396(33.1)	70(33.3)	326(33.0)	1.02(0.74–1.39)	
Chemotherapy					
No	845(70.5)	144(68.6)	701(71.0)	1	1
Yes	353(29.5)	66(31.4)	287(29.0)	1.12(0.81–1.55)	0.83(0.48–1.42)
Stoma					
No	805(76.2)	127(70.2)	678(77.5)	1	1
Yes	251(23.8)	54(29.8)	197(22.5)	**1.46(1.03–2.09)**	1.50(0.93–2.41)
*Health-related characteristics*					
Diabetes					
No	874(84.9)	126(74.1)	748(87.0)	**1**	**1**
Yes	156(15.1)	44(25.9)	112(13.0)	**2.33(1.57–3.47)**	**1.83(1.12–2.98)**
BMI					
Normal weight and underweight	428(36.2)	46(22.5)	382(39.1)	**1**	**1**
Overweight	540(45.7)	94(46.1)	446(45.6)	**1.75(1.20–2.56)**	**2.04(1.26–3.29)**
Obese or morbidly obese	213(18.0)	64(31.4)	149(15.3)	**3.57(2.34–5.45)**	**3.19(1.84–5.54)**

Abbreviations: SD = Standard Deviation; IQR = InterQuartile Range; SES = Socio-Economic Status; BMI = Body Mass Index; OR = Odds Ratio; CI = Confidence Interval.

^1^Odds ratios are derived from univariate logistic regression analyses with the need for dietary support (yes vs. no) as dependent variable and one socio-demographic, cancer-related, or health-related characteristic as independent variable.

^2^ Odds ratios are derived from multiple logistic regression analyses with the need for dietary support (yes vs. no) as dependent variable and all socio-demographic, cancer-related, and health-related characteristics as independent variables.

^3^ Radiotherapy was not included in the multivariable analyses since this variable was interrelated with tumour site with a correlation of >0.70.

Statistically significantly associated variables are printed bold.

### Focus groups

Of the 59 overweight or obese CRC survivors who were invited to participate in the focus groups, sixteen (27.1%) participated in one of three focus groups: one with colon cancer survivors only (n = 5), one with rectal cancer survivors only (n = 6), and one with both colon and rectal cancer survivors (n = 2 rectal, n = 3 colon). Most focus group participants were male (62.5%) and had a lower (50%) or intermediate level (25%) of education. Half of the focus group participants were diagnosed with colon cancer (n = 8). The majority was diagnosed with stage II CRC (68.8%), whereas 18.8% was diagnosed with stage I, and 12.5% with stage III. Half of the focus group participants was overweight and half was obese. Focus group participants did not differ from non-participants with regard to sex, tumour site, tumour stage, BMI, and level of education.

Main themes that emerged during the focus groups included reasons for (not) needing lifestyle support, and needs and preferences for support regarding content, format, timing, context, and provider.

#### Reasons for (not) needing support

CRC survivors reporting a need for lifestyle support stated that they could not initiate and maintain lifestyle changes without appropriate support.

“You actually need support…otherwise I think you won’t succeed…”

Reported reasons for not needing lifestyle support included: already having improved their lifestyle, already having lost weight, and already receiving sufficient support (e.g., support from the home front, or professional support from a dietician). Additionally, perceiving to have a good physical health was mentioned as a reason not to need lifestyle support, whereas having a poor physical health (e.g., as indicated by poor blood values) was mentioned as a reason to need support. The relation between perceived physical health and the need for support was reported to be mediated by readiness for lifestyle change.

#### Reported needs and preferences for support

Content

Participants reported a need for an easily accessible contact person or information point to be able to receive answers to their questions, and to receive information and individually-tailored advice, for example on lifestyle-related issues such as appropriate nutrition, and the use of dietary supplements. In addition to lifestyle-related advice, they also reported a need for individually-tailored advice regarding their disease and treatment-related complaints (e.g., lack of strength, lack of energy, lymphedema, stoma-related problems, and bowel complaints such as too frequent stools and changes in the consistency of the stool). For example, individuals living with a stoma reported a need for advice on appropriate nutrition to reduce problems with their stoma, such as noise and odors caused by certain foods. They generally noted that they needed to receive appropriate answers, information, and advice to be able to make an informed choice on adapting their lifestyle or not.

“I think.. the opportunity should be created.. that you can make choices based on the information provided”

A need for feedback on their lifestyle was mentioned to be able to gain insight into how healthy or unhealthy their lifestyle actually is, and what areas may need improvement. They also reported that having someone monitoring their progress was necessary in order to be able to initiate and maintain lifestyle changes. However, they emphasized that they did not to want to feel forced to change their lifestyle. Instead, they preferred to be able to make an informed, autonomous decision about whether or not to change their lifestyle.

“It has to be without obligations. I want to be able to have to opportunity to say yes or no.”

The importance of contact with fellow-sufferers and involvement of family members during lifestyle support was strongly emphasized. It was perceived to be very important to involve family members, so that they would be triggered to provide social support or to also improve their own lifestyle. Both before and during the focus groups, participants expressed their need for contact with fellow-sufferers. They explicitly stated that one of the reasons they participated in the focus groups was to be able to share their experiences with fellow-sufferers, since they did not have that opportunity in their own social environment. Moreover, they already began to share experiences before the focus group started. They mentioned it was important to share experiences and feelings with fellow-sufferers to be able to learn from each other and to realize that they are not the only ones suffering from CRC cancer and its consequences:

“… I’ve experienced that contact with fellow-sufferers is very important, and it brings things up…things you would normally keep to yourself”

It was mentioned that contact with fellow-sufferers should preferably be in a relatively small group, consisting of fellow-sufferers from the same neighborhood. Preferably, they would like to get in contact with fellow-sufferers with a variety of duration after diagnosis. Both contact with fellow-sufferers with a similar time after diagnosis and contact with long-term survivors was preferred. The latter was preferred because of the feelings of hope a success story from a long-term survivor could provide. They generally preferred to focus on the positive during support, such as the message that there is still a whole life ahead after the diagnosis of cancer.

#### Format and timing

The majority reported that support should be personally-tailored with regard to format, intensity (i.e. frequency and duration of contacts), and timing (i.e. initiation and duration of the support).

“It has to be individually-tailored”.

With regard to preferred format, face-to-face support was generally preferred over digital support (e.g., via the Internet), since digital support was perceived as impersonal. However, a combination of both formats, with a supporting role for digital support, did appeal to participants. Moreover, the use of a leaflet was mentioned as an additional manner to provide information. Furthermore, most participants preferred a support group over individual support. Participants varied with regard to their preferences on the intensity of lifestyle support, which was related to a variety of individual lifestyle goals.

In general, participants mentioned that they would like to have the opportunity to receive support whenever the need for support occurs. They preferred to receive support both during and after treatment, but particularly after completion of treatment. Two periods were preferred in particular: the period from directly after the operation to the first visit after the operation, and the follow-up period, when the frequency of hospital visits decreases and the time between visits increases. Besides, the period directly after diagnosis and before treatment was mentioned as a period during which CRC survivors would like to be informed about the possibility to receive lifestyle support. It was also mentioned that physical recovery was a prerequisite for initiating lifestyle changes, particularly among overweight rectal cancer survivors who more often seemed to report complaints (such as a persistent lack of energy, stoma-related problems, and bowel complaints) limiting their daily functioning after treatment compared with overweight colon cancer survivors. Finally, participants mentioned not to be ready for weight loss until after physical recovery after treatment), which was reported to occur a considerable time (e.g., a year) after completion of treatment.

#### Provider

CRC survivors reported to prefer an easily accessible, experienced counselor to share their experiences and their feelings with, and to answer their questions. They indicated it was important for a counselor to understand what they were going through. They mentioned that support could for example be offered by a gastro-intestinal oncology nurse, an oncology dietician, and/ or a stoma nurse specialist. They also suggested that a contact person from the hospital (e.g., the gastro-intestinal oncology nurse) could play an important role in referring to lifestyle support.

“A good relationship with the nurse or.. a contact person from the hospital…is important, because this person can introduce a dietician … I wouldn’t easily visit a dietician on my own, but if the dietician would be present during a meeting with the nurse or the contact person, and they would suggest to make an appointment with the dietician, I would be more inclined to visit the dietician because I already met him or her”.

#### Context

The hospital was suggested to be a suitable setting to offer or to refer to lifestyle support. Hospital visits with oncology nurses or with oncologists were mentioned to be a suitable context to offer or to refer to lifestyle support. Participants commented that they preferred lifestyle support not to be offered in a commercial setting in which financial profit is the aim of providing support. Moreover, receiving support should preferably not cost too much money, and it should (at least partly) be reimbursed by their health care insurance company. Receiving support should also not cost too much time and effort. Finally, participants mentioned that the fact that some survivors may lack transport to the location of the support should be taken into account.

## Discussion

This study has shown that nearly one fifth of CRC survivors perceived a need for dietary support, and that this need was associated with being younger, living without a partner, having a stoma, having diabetes, and being overweight or obese. Being unable to initiate and maintain lifestyle changes without appropriate support was mentioned as the main reason for needing support, whereas perceiving to have a good physical health was mentioned as one of the reasons not to need support. CRC survivors reported a need for receiving information soon after diagnosis to be able to make an autonomous, informed decision on improving their lifestyle. They preferred to receive individually-tailored lifestyle support in an autonomy-supportive environment, including support to deal with disease and treatment-related complaints and involvement of fellow-sufferers and family.

To our knowledge, our study is the first to report on the proportion of CRC survivors with a perceived need for dietary support to be able to eat healthier, and on characteristics associated with this perceived need. Nevertheless, previous research among CRC survivors[18] and their family members[24], survivors of other types of cancer[34–36], or survivors of mixed types of cancer[37] did evaluate the proportion of cancer survivors with an interest[18] or need[37] to receive additional dietary information[18] or advice[37], and with an interest or willingness to participate in a lifestyle intervention[24, 34–36]. As compared with the proportion of CRC survivors perceiving a need for dietary support in our study (17.5%), these studies reported higher proportions of cancer survivors who were interested in receiving additional dietary information (40%[37]; 91%[24]) or advice (49%)[37], and were interested in or willing to participate in a lifestyle intervention (66%[36] - 98%[18]). This observation is in line with expectations, since it is likely that there are in fact fewer CRC survivors in need for support to be able to change their dietary behaviour than there are CRC survivors who are merely interested in receiving additional dietary information or participation in an intervention. CRC survivors who indicate that they are willing to participate in an intervention or CRC survivors who report an interest or need for receiving dietary information are not necessarily also in need for support. For those in need for dietary information, it is sufficient to receive such information, whereas those in need for support are also in need for additional behavioural counselling to be able to make and maintain dietary changes[22].

Few studies have examined characteristics associated with an interest in receiving dietary information, dietary advice, or participation in an intervention in CRC survivors[24, 18, 37]. Our finding that CRC survivors with overweight and obesity were more likely to report a need for dietary support is in line with findings from a survey (n = 40) from New Zealand[18], which found that CRC survivors with overweight and obesity were more likely to be interested in receiving dietary information than CRC survivors with a BMI in the normal range[18]. Furthermore, our finding that younger CRC survivors were more likely to perceive a need for dietary support is in line with findings from an Italian study[37] among survivors with mixed cancer types and their family members[37], that showed that younger cancer survivors more often reported a need for receiving additional dietary information compared with older cancer survivors[37].

Our finding that CRC survivors with a perceived need for lifestyle support stated that they could not initiate and maintain lifestyle changes without appropriate support, suggests that a lack of self-efficacy to make lifestyle changes is an important reason for needing support. This confirms the results of previous research on the relation between self-efficacy and making lifestyle changes[25, 38, 39]. These findings suggest that promotion of self-efficacy is an important target in lifestyle interventions for cancer survivors.

Our study also provided insight into reasons for not needing lifestyle support among CRC survivors. Our finding that perceiving to have a good current physical health was related to not needing support because of a lack of readiness for lifestyle change, suggests that promoting readiness to change may influence the need for lifestyle support. Information provision on lifestyle and body weight recommendations for cancer survivors and provision of individually-tailored feedback on current lifestyle may promote readiness for improving lifestyle, and uptake of support in CRC survivors who do not meet lifestyle and body weight recommendations.

To date, few studies have been conducted on preferences for dietary and/ or physical activity support among CRC survivors[25, 24, 19, 18]. Our finding that CRC survivors preferred to receive individually-tailored information and advice is in line with other studies on preferences for lifestyle support among CRC survivors[24, 19]. Since our focus groups also show that most participants prefer a support group over individual support, our findings suggest that a combination of both formats may be suitable for use in CRC survivors. For example, group lifestyle counselling sessions could be supplemented with computer-tailored individual counselling.

Our assumption that CRC survivors were more likely to make sustainable lifestyle changes after recovery from treatment was supported by our focus group results on preferred timing of support. Our focus group participants reported to particularly need lifestyle support after completion of treatment during follow-up. Additionally, they preferred to receive information on the possibility of receiving support in the period directly after diagnosis and before treatment. These findings on perceived optimal timing of support are in line with previous studies among CRC survivors[24, 19], and suggest that multiple ‘teachable moments’ can be identified.

As in other studies exploring preferences for lifestyle support among CRC survivors[25, 19], disease and treatment-related complaints were reported to limit the ability to make lifestyle changes in our study. In line with these studies, the results of our focus groups suggest that dealing with complaints as a consequence of CRC and its treatment should be an integral part of lifestyle support for CRC survivors, and should include information provision about potential disease and treatment-related complaints, and individually-tailored advice regarding complaints that arise throughout the treatment process and thereafter. Furthermore, our results suggest that it should be explored during lifestyle support how lifestyle can be improved while acknowledging and taking into account individual disease and treatment-related complaints. Moreover, lifestyle support should also include information provision about the effect of lifestyle on such complaints. For example, it should include information provision on the influence of nutrition on the consistency of the stool in case of diarrhoea. During our focus groups, rectal cancer survivors and survivors with a stoma particularly reported disease and treatment-related complaints. The results of our focus groups suggest that rectal cancer survivors more often experienced such complaints and experienced more severe complaints as compared with colon cancer survivors. Previous research confirms that bowel problems are generally more common and problematic in rectal cancer survivors compared with colon cancer survivors[16]. In addition, rectal cancer survivors more often have a stoma, and having a stoma can particularly lead to problems in individuals with overweight or obesity.

Our findings on the perceived importance of involvement of family members during lifestyle support, confirm the findings of other studies examining preferences for lifestyle support among CRC survivors[25, 24]. In addition, our finding that CRC survivors expressed a preference for contact with fellow-sufferers, is in line with a study that found that contact with fellow-sufferers was considered to be helpful to CRC survivors[19].

Our findings on preferences for lifestyle support provide guidance on which Behavior Change Techniques (BCT’s) could be used to promote adherence to lifestyle and body weight recommendations in CRC survivors[40]. Our finding that CRC survivors preferred to receive feedback about their current lifestyle suggests that the BCT ‘feedback on behaviour’[40] may be used to promote adherence. Furthermore, our finding that participants preferred someone to monitor their progress during support suggests that the BCT ‘review behavior goal(s)’ should be involved in lifestyle support[40]. Finally, our finding that CRC survivors preferred involvement of fellow-sufferers and the home front suggests that the BCT ‘social support’ could be used to promote adherence. These BCT’s have all been associated with significant intervention effects [41, 42].

Our findings on the content of preferred lifestyle support are in agreement with the Self-Determination Theory (SDT)[43]. Our findings confirm that autonomy, relatedness, competence, and an autonomy supportive environment are determinants of lifestyle changes among CRC survivors. Our finding that CRC survivors did not want to feel forced to change their lifestyle, illustrates their need for an autonomy supportive environment. They expressed a need for autonomy (i.e., feeling volitional and feeling choice and responsibility for one's behavior) by stating their preference to be able to make a conscious, autonomous decision about whether or not they wanted to change their lifestyle. This need for autonomy was also illustrated in another study examining preferences for support among cancer survivors[24]. Participants’ need for relatedness (i.e., feeling understood, cared for and valued by significant others) was illustrated by their preference to involve fellow-sufferers and the home front in lifestyle support. Finally, their need for competence (i.e., feeling that one can accomplish a behavior or reach a certain goal) was illustrated by our finding that a lack of self-efficacy for achieving and maintaining a healthy lifestyle was reported to be an important reason to need lifestyle support. Since the results of our focus groups seem to fit the SDT, lifestyle interventions based on SDT seem appropriate to promote lifestyle changes in CRC survivors. Although SDT-based interventions have shown promising long-term effects[44–46], they have not yet been applied in cancer survivors. Future research should be conducted to further explore the suitability SDT-based support tailored for CRC survivors.

### Strengths and limitations

Strengths of our survey include a large population-based sample and a high response rate, which are mainly attributable to data collection through the well-established PROFILES registry as part of a longitudinal study[26]. The use of a mixed methods-design is also a strength since using quantitative and qualitative research methods complementary allowed us to study the need for dietary support among CRC survivors in more detail.

Several limitations need to be taken into account while interpreting the results of this study. Our findings should be interpreted with caution for stage IV CRC survivors as this particular subgroup was underrepresented in our study. When interpreting our findings, it should be taken into account that receiving information on lifestyle and body weight recommendations for cancer survivors was not part of clinical care by the time of the study. It is expected that if CRC survivors would be informed about lifestyle and body weight recommendations for cancer survivors, a larger proportion would probably want to improve their diet and a larger proportion would perceive a need for dietary support. Furthermore, the number of cancer survivors with a need for dietary support could have been underestimated due to a tendency towards socially desirable answers. It is possible that participants did not want to admit they were in need for dietary support. Additional analyses among respondents vs. non-respondents confirm a possible underestimation of those in need for dietary support, given that mean age in non-respondents was lower compared with those excluded from the population for analyses and our finding that younger age was associated with a need for dietary support (data not shown). Ideally, we would have recruited focus group participants from the survey participants. Since this was not possible, we recruited focus group participants from the COLON-study[31], a study with a generally comparable study sample.

### Recommendations

Our results suggest that oncology health care professionals should routinely offer information on lifestyle and body weight recommendations for cancer survivors to enable CRC survivors to make an informed decision on improving their lifestyle. Our study provides guidance on which CRC survivors particularly perceive a need for dietary support, and suggests that support should particularly be offered to younger CRC survivors, those living without a partner, those with a stoma, those with diabetes, and those with overweight or obesity. Appropriate lifestyle support should be offered to CRC survivors throughout the treatment process and thereafter, preferably from directly after diagnosis onwards. Findings from our study on preferences for lifestyle support can be used to tailor lifestyle support to the needs and preferences of CRC survivors in order to promote uptake, adherence, and effectiveness of such support[47]. These findings suggest that support should be individually-tailored, autonomy-supportive, include how to deal with disease and treatment-related complaints, and should involve fellow-sufferers and the home front. For example, lifestyle support could be tailored to CRC survivors’ preference for autonomy-supportive support by providing multiple options and allowing choice (e.g., on ways to improve diet quality) instead of prescribing a particular diet.

Future research should be conducted on how to promote adherence to lifestyle and body weight recommendations in CRC survivors who do not meet (one or more) recommendations, and should take into account CRC survivors’ readiness to change and their perceived need for support. Different approaches are required to promote adherence in CRC survivors who differ with regard to their readiness to change and their perceived need for support. A first step to promote adherence to lifestyle and body weight recommendations in CRC survivors, irrespective of their readiness to change and their perceived need for support, would be to increase awareness and knowledge on these recommendations (e.g., by routinely providing information on these recommendations).

To be able to gain more insight into the optimal strategy to achieve sustained lifestyle improvement in CRC survivors, further research should focus on identification of effective intervention components. According to the first step in systematic intervention planning (i.e., needs assessment)[23], more knowledge is needed on determinants of adherence to lifestyle and body weight recommendations in CRC survivors to be able to select potential effective intervention components for application in CRC survivors. Future research should be conducted to assess the effectiveness of lifestyle support tailored to the needs and preferences of CRC survivors, particularly on the long-term.

### Conclusions

This study has shown that nearly one fifth of CRC survivors perceived a need for dietary support to be able to eat healthier and suggests that dietary support should particularly be offered to younger CRC survivors, those living without a partner, those with a stoma, those with diabetes, and those with overweight or obesity. Results suggest that information about lifestyle and body weight recommendations should be provided after diagnosis to enable CRC survivors to make an autonomous, informed decision on whether or not they want to change their lifestyle. In addition, results suggest that lifestyle support for CRC survivors should be individually-tailored, autonomy-supportive, include how to deal with disease and treatment-related complaints, and should involve fellow sufferers and the home front. Findings from our study can be used to better identify CRC survivors in need for dietary support, and to tailor lifestyle support to their needs and preferences in order to promote uptake, adherence, and effectiveness of support.
